# Antioxidant and Anti-Inflammatory Properties of Four Native Mediterranean Seagrasses: A Review of Bioactive Potential and Ecological Context

**DOI:** 10.3390/md23050206

**Published:** 2025-05-12

**Authors:** Marzia Vasarri, Lucia De Marchi, Carlo Pretti, Emanuela Barletta, Donatella Degl’Innocenti

**Affiliations:** 1 Department of Experimental and Clinical Biomedical Sciences, University of Florence, Viale Morgagni 50, 50134 Florence, Italy; emanuela.barletta@unifi.it (E.B.); donatella.deglinnocenti@unifi.it (D.D.); 2 Department of Veterinary Sciences, University of Pisa, Viale delle Piagge 2, 56124 Pisa, Italy; lucia.demarchi@unipi.it (L.D.M.); or pretti@cibm.it (C.P.); 3 Interuniversity Center of Marine Biology and Applied Ecology “G. Bacci” (CIBM), Viale N. Sauro 4, 57128 Leghorn, Italy

**Keywords:** native Mediterranean plants, antioxidant, anti-inflammatory, seagrass, *Posidonia oceanica*, *Cymodocea nodosa*, *Zostera marina*, *Zostera noltii*

## Abstract

This review provides current knowledge of the potential benefits of native Mediterranean seagrasses for human health, specifically focusing on their anti-inflammatory and antioxidant properties. The four main species examined—*Posidonia oceanica*, *Cymodocea nodosa*, *Zostera marina*, and *Zostera noltii*—are integral components of marine ecosystems, providing essential habitats and supporting biodiversity. Recent studies highlight their rich bioactive compounds that show significant therapeutic potential against oxidative stress and chronic inflammation, which are prevalent in various health disorders. This overview synthesizes the current literature, emphasizing the mechanisms through which these seagrasses exert their beneficial effects. Furthermore, it addresses the environmental implications of the excessive use and abuse of conventional anti-inflammatory drugs, advocating for a shift towards natural alternatives derived from marine resources. By exploring the bioactivity of these Mediterranean seagrasses, research here collected underscores the importance of integrating marine plants into health and wellness strategies, thereby promoting both human health and ecosystem sustainability. This exploration not only enriches the understanding of their applications on human health but also stimulates further research in this promising field, paving the way for innovative approaches to combat chronic diseases and support environmental conservation.

## 1. Introduction

Inflammation is a crucial and complex biological process that plays a fundamental role in the body’s defense mechanism. It occurs as a response to injury, infection, or exposure to harmful stimuli, serving to protect the body by eliminating the source of damage, removing dead or damaged cells, and promoting tissue repair [1]. The acute inflammatory response is essential for healing and maintaining health; however, when inflammation becomes persistent or uncontrolled, it can have detrimental effects [2].

Chronic inflammation has been linked to the development and progression of numerous diseases, including cardiovascular disease, diabetes, cancer, and neurodegenerative disorders, among many others. Therefore, regulating the inflammatory process is vital for developing strategies to prevent and treat these chronic conditions, emphasizing the importance of maintaining a balanced immune response for overall health [3].

A key factor intertwined with inflammation is the production of free radicals: unstable molecules that contain unpaired electrons that make them highly reactive. Common free radicals, such as reactive oxygen species (ROS) like superoxide anion, hydroxyl radical, and hydrogen peroxide, are naturally produced within the cells during metabolic processes, especially in the mitochondria during energy production [4].

The body’s antioxidant defense system is essential in protecting cells, DNA, and tissues from the damaging effects of these reactive molecules. This system includes endogenous antioxidants, such as enzymes like superoxide dismutase (SOD), catalase (CAT), glutathione peroxidase (GPx), and glutathione reductase (GR), which are produced internally to neutralize ROS. Additionally, exogenous antioxidants obtained from the diet, including vitamin C, vitamin E, polyphenols, flavonoids, and carotenoids, contribute significantly to reducing oxidative stress. Maintaining a robust antioxidant system is essential for overall health and for preventing diseases associated with oxidative damage [4]. However, while low to moderate levels of ROS play essential roles in cell signaling, immune defense, and maintaining homeostasis, excessive ROS production can cause oxidative damage to lipids, proteins, and DNA. This damage impairs cellular function and integrity, potentially leading to various health issues if left unchecked [5,6]. Indeed, oxidative stress arises when there is an imbalance between the production of free radicals and the body’s ability to neutralize them with antioxidants. This imbalance results in damage to cellular components and contributes to aging and the development of numerous diseases. During inflammation, immune cells such as macrophages and neutrophils generate large amounts of ROS to destroy pathogens. However, excessive or prolonged ROS production can also harm host tissues, perpetuating inflammation and creating a vicious cycle of oxidative stress.

The relationship between inflammation, free radicals, and oxidative stress is complex and bidirectional, each process influencing the other. Inflammation can increase ROS production, leading to oxidative stress, which in turn can activate further inflammatory pathways in a persistent cycle [7,8]. This interplay is central to many chronic diseases, highlighting the importance of antioxidant defenses and anti-inflammatory strategies. Understanding the roles of inflammation, free radicals, and oxidative stress is crucial for developing therapies aimed at reducing oxidative damage and controlling chronic inflammation. A comprehensive approach that addresses these interconnected processes is essential for preventing and managing a wide array of health conditions linked to inflammation and oxidative damage [4].

### 1.1. Seagrasses: Marine Flowering Plants, Ecosystem Functions, and Societal Significance

Seagrasses, named for their long green leaves, are often mistaken for algae; however, they are more closely related to land plants [9]. These fascinating marine flowering plants originated from terrestrial ancestors that made their way back to the ocean approximately 70–100 million years ago [10,11]. Seagrasses form dense underwater meadows that are among the most productive ecosystems on Earth, serving as crucial carbon sinks and providing habitat and sustenance for a diverse array of marine life, rivaling the biodiversity found in coral reefs [12,13,14]. These underwater meadows play a vital role in maintaining the health and sustainability of marine environments, significantly contributing to biodiversity, coastal stability, and water quality [15].

For millennia, seagrass meadows have been intertwined with human culture, representing a natural resource that has sustained humanity throughout history [16,17]. Over the last 180,000 years, these seagrass meadows have provided spiritual, medicinal, and material benefits, serving as a food source for both humans and marine life, and influencing various cultural practices and local economies. Although many traditional customs have been lost or modified, recent rediscoveries and innovations, such as the use of seagrasses in sustainable food production and construction material, demonstrate the ongoing connection between these ecosystems and human communities. The importance of sustainable harvesting practices is highlighted to prevent overexploitation and promote conservation, linking the health of seagrass ecosystems to broader environmental and social well-being. This connection supports the integration of seagrasses into modern economic practices to achieve the Sustainable Development Goals (SDG, also known as the Global Goals), adopted by the United Nations in 2015 to achieve a better and more sustainable future for all by 2030 [16].

**Figure 1:**
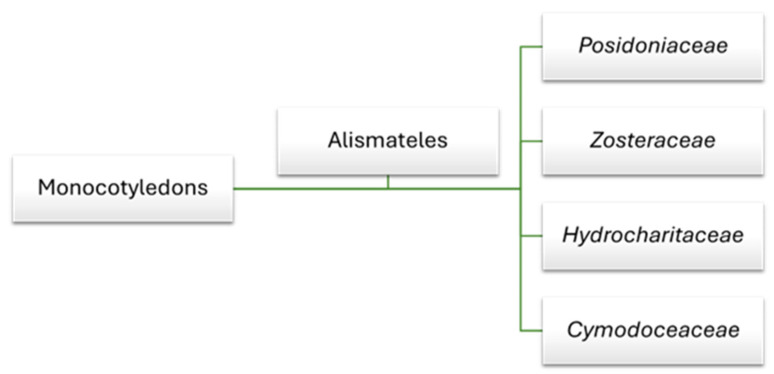
Seagrass family within the order Alismatales in the monocot clade.

Globally, there are about 60 species of marine flowering plants, with only 4 considered native to the Mediterranean Sea [18]. These species belong to four families: *Posidoniaceae*, *Zosteraceae*, *Hydrocharitaceae*, and *Cymodoceaceae*, which are all classified within the order Alismatales and the clade of monocotyledons (Figure 1).

The four native species found in Mediterranean waters are *Posidonia oceanica*, *Cymodocea nodosa*, *Zostera marina*, and *Zostera noltii* [19]. Among these, *Posidonia oceanica* and *Cymodocea nodosa* play a fundamental role as structuring species within marine flowering plant communities. These plants not only provide essential habitat for a variety of marine organisms but are also capable of hosting various epiphytic algae, thereby contributing to the biodiversity of marine ecosystems [20,21]. Their presence is crucial for the health of coastal environments, performing vital ecological functions and supporting marine life [12,22].

#### 1.1.1. *Posidonia oceanica*

**Figure 2:**
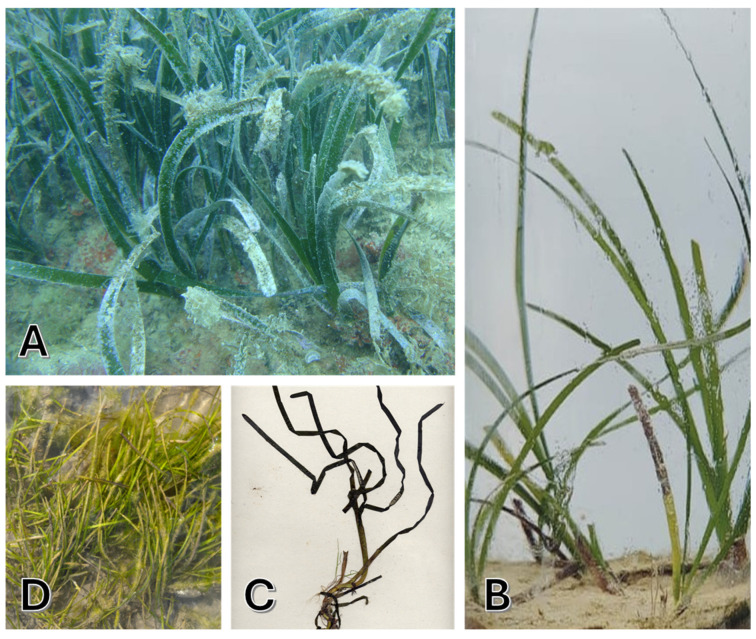
Representative images of the native Mediterranean plants: (**A**) *Posidonia oceanica*, (**B**) *Cymodocea nodosa*, (**C**) *Zostera marina*, and (**D**) *Zostera noltii.*

*P. oceanica*, commonly known as “Neptune Grass”, is an endemic species of the Mediterranean Sea that thrives in clear, shallow waters, up to 25–30 m deep, and occasionally up to 40 m in insular areas [23] (Figure 2A). Recognized as one of the planet’s oldest and most remarkable organisms, *P. oceanica* can live for nearly 100,000 years [24]. Its extremely slow growth and remarkable longevity enable the formation of seagrass meadows that can persist for millennia. However, this slow growth also renders it susceptible to environmental stresses, meaning that recovery from any damage can take decades or even centuries [25]. While *P. oceanica* can tolerate some fluctuations in temperature, thriving best between 10–25 °C, prolonged exposure to extreme conditions can adversely affect its health and vitality [26]. This species is found exclusively in Mediterranean waters, where it creates barriers of compact sediments that help stabilize marine ecosystems. Its broad, elongated leaves grow in bundles attached to vertical rhizomes that spread horizontally, making the plant easily identifiable. Flowering occurs infrequently; its propagation primarily occurs through vegetative means via rhizome branching, further increasing the species’ vulnerability to human activities and climate change [27].

#### 1.1.2. *Cymodocea nodosa*

*C. nodosa*, known as “seahorse grass”, is a seagrass species that grows in warm waters of the Mediterranean, the Canary Islands, and along the North African coast up to southern Portugal (Figure 2B). It prefers depths between 0–60 m. The leaves are 2–4 mm wide and 10–45 cm long, and the white or pink rhizomes grow vertically and branch horizontally, facilitating the colonization of bare substrates. The flowering, which occurs rarely between May and August, produces seeds that are larger than those of *Z. marina* [19]. Considered a pioneer species, *C. nodosa* is more tolerant of environmental fluctuations compared to *P. oceanica* [28,29] and often precedes the climax communities of *P. oceanica* [30,31]. It thrives best in shallow coastal waters, estuaries, and lagoons [32], preferring muddy or sandy substrates up to 35 m deep. It requires good illumination and tolerates moderate water movement, with an optimal temperature range between 10–32 °C, making it relatively resilient to future climate changes [33,34].

#### 1.1.3. *Zostera marina* and *Zostera noltii*

*Z. marina*, commonly referred to as “greater eelgrass”, a perennial marine plant essential for the coastal ecosystems of the Northern Hemisphere, is found in shallow waters at 10–25 °C (Figure 2C). In the Mediterranean, *Z. marina* grows in small colonies in areas of low salinity. Its stems can reach up to 1.5 m, and the leaves have an average length of 20–35 cm. Reproduction occurs both vegetatively and sexually, but fruiting is rare [19]. In recent years, *Z. marina* has declined in the Mediterranean due to pollution and anthropization. Seagrass meadows of *Z. marina* are vital habitats for many marine species, and their disappearance threatens biodiversity. The conservation of this plant is crucial for the health of coastal marine ecosystems [14,35]. Interestingly, research conducted in 2016 by Olsen et al. reported for the first time the sequencing of the whole genome of the seagrass *Z. marina*, becoming the first angiosperm to be sequenced [36].

*Z. noltii*, or “dwarf eelgrass”, is a Mediterranean marine plant forming dense beds in intertidal muddy sands (Figure 2D). Notable for its tolerance to desiccation, it thrives in areas with significant tidal changes, unlike *Z. marina*. Its leaves, 5 to 25 cm long and 0.5–2 mm wide, are anchored to a horizontal rhizome, providing stability in coastal environments. The plant’s hermaphroditic flowers produce seeds likely dispersed by waterfowl, aiding its propagation. *Z. noltii* thrives in shallow waters, tolerating temperatures up to 38 °C, which enhances its adaptability. This resilience makes it vital for marine biodiversity, offering habitat for various organisms and contributing to coastal stability and ecosystem sustainability. In essence, *Z. noltii* is crucial for the health of marine environments and coastal protection [14,37].

### 1.2. Ecological Functions of Seagrasses and Their Secondary Metabolites

Seagrasses such as *P. oceanica*, *C. nodosa*, *Z. marina*, and *Z. noltii* are known to produce a diverse array of secondary metabolites that serve as vital defense mechanisms during stressful conditions. These compounds help protect the plants from oxidative stress caused by environmental challenges like UV radiation, temperature fluctuations, and water pollution. These secondary metabolites, like polyphenols, flavonoids, and polysaccharides, have been reported to possess a wide range of beneficial properties, including anticancer, antifungal, anti-inflammatory, antimicrobial, antiviral, antidiabetic, antimalarial, antioxidant, anti-aging, and cytotoxic effects. Moreover, these seagrass species are considered effective in the prevention of various human diseases, highlighting their potential as valuable sources for therapeutic applications [38,39].

## 2. Inflammation and Oxidative Stress: The Power of Marine Plants and Their Potential Environmental Impact

Since ancient times, early societies have used crude extracts of plants to treat infections, inflammation, pain and a variety of other ailments [38]. In recent decades, attention has been paid to marine herbal medicine to develop new therapeutic approaches or for other medical purposes [40]. In fact, studies worldwide have shown that several crude extracts derived from marine plants contain innovative bioactive compounds that can benefit health and can be used in medicine or food [41,42,43].

Research indicates that marine plants are abundant in micronutrients and secondary metabolites such as polyphenols, flavonoids, and carotenoids, which are essential in the prevention and/or treatment of chronic diseases, and in the promotion of human health [43,44]. Among these bioactive compounds, some are recognized for their therapeutic potential against inflammation and oxidative stress, two important aspects of many chronic diseases that are nowadays a global economic and health challenge [45,46].

Chronic inflammation and oxidative stress are a common factor of a wide range of disorders and diseases. Inflammatory processes not only affect short-term health, but also contribute to an overall decline in long-term health [47,48], negatively affecting health status and increasing susceptibility to various diseases that are not necessarily inflammatory. Chronic inflammation is characterized by a persistent immune response that can damage tissues and organs, while oxidative stress results from an excess of free radicals in the body, leading to cell damage and accelerated ageing [2,49]. Therefore, curbing the inflammatory process and oxidative stress is an important global goal in both medicine and prevention.

Currently, among the various methods to address inflammation, non-steroidal anti-inflammatory drugs (NSAIDs) are among the most widely used. NSAIDs are commonly employed to relieve pain, reduce inflammation, and lower fever [50]. While these drugs are effective for many patients, they are not universally suitable and can lead to a range of side effects [51,52,53]. Furthermore, the ease of access to NSAIDs, often available over the counter, encourages self-medication and can contribute to their excessive use and abuse [54]. This widespread drug consumption raises significant concerns regarding environmental contamination, as NSAIDs frequently enter aquatic ecosystems through wastewater effluents and household waste, posing risks to both wildlife and human health [55,56,57,58].

In light of these challenges, there is a growing interest in exploring natural products as alternative therapies. Indeed, natural substances are often renewable resources and generally exhibit a lower toxicity profile compared to conventional drugs [46]. This shift from chemicals to naturals can help mitigate the side effects associated with conventional treatments. Moreover, many natural compounds and/or phytocomplexes possess multiple mechanisms of action, which may enhance the efficacy of existing treatments.

**Figure 3:**
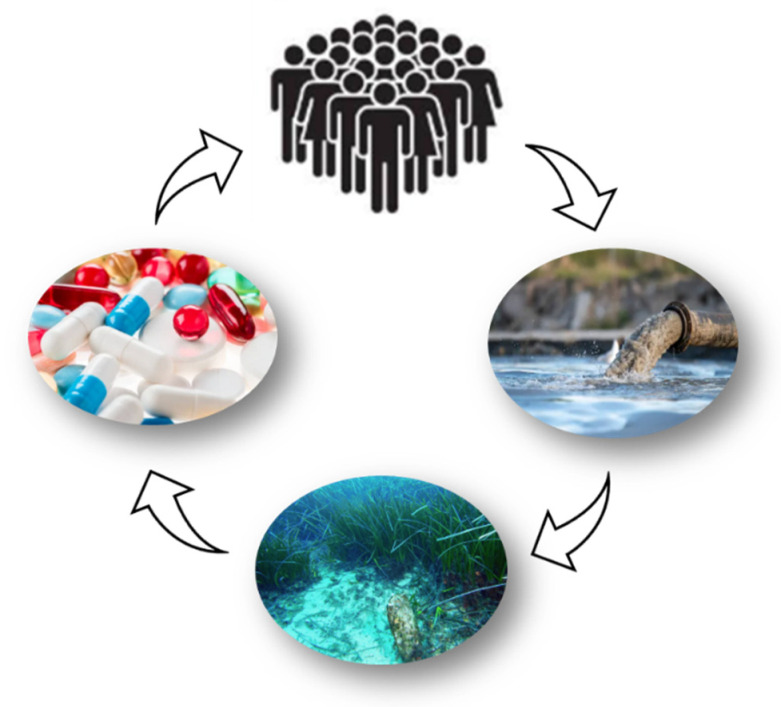
Marine pharmaceuticals: a virtuous cycle for human health and the preservation of marine ecosystems. The effects of pharmaceuticals extend far beyond the human body. All medications are eventually excreted from the body. Active ingredients or mixtures of metabolites are expelled through feces and urine. These substances flow into sewage treatment, which often struggles to eliminate the pharmaceutical residues that inevitably end up in the planet’s waters.

The exploration of marine-derived therapies presents an opportunity not only to address health concerns but also to reduce environmental pollution, aligning with the principles of a circular economy: utilizing resources from the sea that can ultimately return to the marine environment in a sustainable manner (Figure 3). In this context, the identification and application of natural marine substances with anti-inflammatory and antioxidant properties are becoming increasingly important [46,59,60,61].

By harnessing the potential of these compounds, we can effectively tackle health issues related to chronic inflammation and oxidative stress while simultaneously minimizing the ecological impact of pharmaceutical waste. This approach not only supports individual health but also promotes environmental sustainability, paving the way for a more responsible and holistic healthcare system [16].

As the Mediterranean native plants are poorly known in terms of their bioactivity, this review aims to provide a comprehensive analysis of the scientific evidence on their anti-inflammatory and antioxidant properties. By synthesizing the current literature, we will highlight the mechanisms through which these marine plants exert their beneficial effects, as well as their potential applications for human health. This exploration will not only enhance our understanding of the therapeutic potential of marine plants native to the Mediterranean but will also stimulate further research in this promising and rapidly expanding field. As we continue to explore the health benefits of these extraordinary plants, we may discover new strategies to address chronic diseases and promote general well-being.

## 3. Exploring the Antioxidant and Anti-Inflammatory Benefits of the Native Mediterranean Seagrasses

### 3.1. Posidonia oceanica Bioactivity

Since ancient times, the marine plant *P. oceanica* has been valued not only for its ecological role but also for its remarkable therapeutic properties. The ancient Egyptians, for example, used it to treat various ailments, demonstrating a long tradition in folk medicine [17]. Historical documentation confirms the use of *P. oceanica* to alleviate inflammation, acne, colitis, and respiratory infections, making it a valuable remedy for multiple pathologies [62,63]. Currently, contemporary research is rediscovering and delving into its potential, highlighting its beneficial properties and contributing to a renewed interest in a plant that has much to offer for human health and well-being.

This paragraph will analyze in detail the antioxidant and anti-inflammatory effects of *P. oceanica*, which could open new avenues in the treatment of various diseases.

For the first time, Gokce et al. (2008) described the antioxidant activity of a hydroalcoholic extract of *P. oceanica* leaves in an alloxane-induced diabetic Wistar albino rat model. The authors showed that oral administration of the extract (150 and 250 mg/kg body weight) led to a decrease in blood glucose and in a reduction in oxidative stress markers in the livers of diabetic rats, accompanied by increased levels of glutathione, GPx, SOD, CAT, nitric oxide (NO), and malondialdehyde (MDA) [63].

In the last decade, extracts of *P. oceanica* have attracted increasing scientific attention for their potential in the field of human health. In this context, *P. oceanica* stands out as a significant source of bioactive compounds, particularly polyphenols. These compounds, including phenolic acids and flavonoids, have been recognized for their ability to neutralize free radicals, reduce oxidative stress, and protect cells from damage caused by external agents [17].

In the research conducted by Messina et al. (2021), HPLC analysis revealed significant differences in the phenolic compound content of *P. oceanica* leaves, depending on their physiological state. Green leaves, rich in chlorophyll and photosynthetically active, show a higher polyphenol content compared to brown leaves and exhibit better antioxidant potential. This suggests that the harvesting and analysis of *P. oceanica* leaves could provide valuable insights for nutritional and therapeutic applications [64]. Additionally, extraction conditions, such as the grinding method and the type of solvent used, have a significant impact on the yield and antioxidant activity of the extracts. For instance, the Gd-E4 extract (obtained from green leaves dried at 60 °C for two days) showed the highest yield, with a polyphenols content of 19.712 ± 0.496 mg gallic acid equivalents (GAE)/g, as well as superior biological activity, evidenced by a DPPH IC_50_ value of 0.090 µg/µL. Particularly, HPLC analysis revealed chicoric acid as the most abundant phenolic compound in Gd-E4 (4991.813 µg/g). These findings underscore the importance of optimizing drying parameters to preserve heat-sensitive phenolic compounds, thereby maximizing extract quality and bioactivity [64]. Furthermore, the authors tested the photoprotective capacity of *P. oceanica* green leaf (Gd-E4) extracts in human skin fibroblasts (HS-68 cells) subjected to UV-induced oxidative stress. The reported results suggest that *P. oceanica* Gd-E4 extracts (0.15–1.5 µg/mL) induce significant protection against oxidative stress and mortality associated with UV exposure. In this context, the potential cosmetic applications of compounds extracted from *P. oceanica* appear promising, especially in the prevention of skin aging [64]. Additionally, the analysis of the ethanolic extract of *P. oceanica* leaves (PEE) by Cornara et al. (2018) highlighted chicoric acid as the main component of the extract (55.8 ± 7 mg/g dry weight in the PEE). Other major PEE compounds identified by HPLC-MS and tandem MS/MS were flavonoids, including procyanidin C2, procyanidin B2, isorhamnetin-3-*O*-glucoside, quercetin-3-*O*-glucoside, quercetin-3-*O*-malonylglucoside, and isorhamnetin-3-*O*-malonylglucoside. These compounds possesses remarkable in vitro antioxidant properties. The PEE showed an IC_50_ value of 32 ± 2 μg/mL in the DPPH radical scavenging assay, corresponding to 6.5 mM (1.14 mg/mL) ascorbic acid equivalents (AAE) [65]. The authors attributed the antioxidant activity of the extract rich in chicoric acid and flavonoids to its ability to stimulate human skin fibroblasts activity (20 µg/mL of PEE), promoting the collagen synthesis essential for maintaining skin elasticity and reducing signs of aging. The extract also demonstrated potential skin whitening effects by inhibiting the in vitro activity of the mushroom enzyme tyrosinase, with an estimated IC_50_ of 14.7 µg/mL of the PEE. Additionally, the PEE at a concentration of 50 µg/mL reduced melanin content in melanoma cells by approximately 50% within 72 h, suggesting a multifunctional approach to address hyperpigmentation problems [65]. The lipolytic effects observed by measuring the glycerol released by adipocytes following the triglyceride degradation indicate that the PEE (10–200 µg/mL) could have applications in the treatment of cellulite, improving skin texture.

By delving deeper into the focus on bioactive compounds in the research by Messina et al. (2021) and Cornara et al. (2018), it is highlighted that phenolic components (primarily chlorogenic acid) are fundamental in the bioactive profile of *Posidonia oceanica* extracts, with significant therapeutic and cosmetic potential, although the authors do not describe a molecular mechanism through which the extracts act on this process.

The antioxidant activity of *P. oceanica* was also reported by Piva et al. (2017), who evaluated a hydroalcoholic leaf extract (PO) rich in polyphenols using the DPPH assay [66]. The *P. oceanica* extract exhibited a total phenolic content of 711 mg GAE/g extract, and demonstrated an antioxidant capacity with an IC_50_ value of 72.42 ± 22.9 mg/L [66]. Additionally, Kevrekidou et al. (2024) investigated the methanolic extract of *P. oceanica* living leaves (LP). The total phenolic content of the LP extract was 222.80 ± 13.99 mg GAE/g dry weight. This study identified chicoric acid as the main polyphenol present in the extract (7.059%). The antioxidant activity was assessed using multiple methods, including the DPPH assay (IC_50_ value of 8.2 ± 0.8 μg/mL), ABTS radical scavenging assay (IC_50_ value of 1.6 ± 0.9 μg/mL), hydroxyl radical scavenging assay (IC_50_ value of 267 μg/mL), superoxide anion radical scavenging assay (IC_50_ 71.0 ± 2.0 μg/mL), and reducing power assay (19.5 ± 0.6 mg/mL) [67].

These data from Piva et al. (2017) and Kevrekidou et al. (2024) collectively confirm that *P. oceanica* possesses substantial antioxidant activity, largely driven by its high polyphenolic content, including chicoric acid. Its efficacy across multiple radical scavenging assays underscores its potential as a natural source of antioxidants.

The role of *P. oceanica* in counteracting oxidative stress and its anti-inflammatory properties was studied in a lipopolysaccharide (LPS)-stimulated murine macrophage model (RAW264.7 cells). The tested hydro-ethanolic extract from *P. oceanica* leaves (POE) contained 3.6 ± 3 mg GAE/mL of total polyphenols. Using UPLC analysis, the authors characterized the extract, revealing a large amount of catechins and smaller quantities of other polyphenols (gallic acid, chlorogenic acid, ferulic acid, epicatechin) [68]. Additionally, the POE exhibited a radical scavenging activity in the DPPH assay and antioxidant activity in the FRAP assay, with values of 11.0 ± 0.7 mg AAE/mL and 0.9 ± 0.2 mg AAE/mL, respectively.

The POE (2.88 µg GAE/mL) was shown to provide non-toxic protection against LPS-induced damage by reducing intracellular ROS levels and cytotoxicity and by modulating inflammatory mediators such as NO, inducible nitric oxide synthase (iNOS), and cyclooxygenase-2 (COX-2) [68]. Furthermore, it was shown that the POE prevented the phosphorylation and activation of NF-κB, a crucial signaling pathway in the inflammatory response, and its upstream pathways, ERK1/2 and Akt [68]. The implications of these findings are significant: they not only contribute to understanding *P. oceanica* as an anti-inflammatory agent but also highlight its potential role in the co-treatment of chronic diseases associated with inflammation. In the work of Micheli et al. (2021), a polyphenol-rich hydroalcoholic extract of *P. oceanica* leaves (POE) showed a radical scavenging activity of 1.2 ± 0.04 mg AAE/mL (tested by a DPPH assay) and an antioxidant activity of 0.24 ± 0.05 mg AAE/mL (tested by a FRAP assay). The authors tested the POE in an in vivo experimental model of acute inflammatory pain in CD-1 mice. The POE demonstrated a dose-dependent effect when orally administered (10–100 mg/kg body weight), reducing inflammatory and oxidative markers, increasing the pain threshold, and decreasing edema formation [69]. The POE (100 mg/kg body weight) has been shown to decrease myeloperoxidase (MPO) activity and levels of inflammatory cytokines, such as IL-1β and TNF-α, in tissues, underscoring its ability to modulate inflammatory responses. This study represents a significant step, as it is the first to provide pharmacological evidence regarding the ability of *P. oceanica* to alleviate inflammatory pain in vivo [69].

Based on these findings, the research conducted by Micheli et al. (2024) examined the effect of POE on C57BL/6 murine model of psoriasis-like skin lesions induced by Imiquimod for 5 days. The antioxidant activity of POE was measured in vitro by DPPH and FRAP assays, with values of 1.1 ± 0.2 mg AAE/mL and 0.13 ± 0.07 mg AAE/mL, respectively. The oral administration of the POE (100 mg/kg body weight) showed promising results, significantly reducing the PASI score (Psoriasis Area Severity Index) and the histological features characteristic of psoriasis, such as hyperkeratosis [70]. The *P. oceanica* extract inhibited the expression of key inflammatory cytokines, such as TNF-α, IL-17A, and IL-23, suggesting that this plant extract not only modulates inflammatory signals but may also prevent the activation of the NF-κB pathway, which is fundamental in the pathogenesis of psoriasis. Furthermore, the reduction of the plasma levels of lipocalin-2, a potential therapeutic target for psoriasis, further highlights the modulatory action of the *P. oceanica* extract [70].

Both the in vitro and in vivo results consistently indicate that the POE exerts antioxidant and anti-inflammatory effects through modulation of key signaling pathways such as NF-κB and upstream kinases. The in vitro data elucidate molecular mechanisms at the cellular level, including ROS scavenging and cytokine suppression, while the in vivo studies demonstrate tangible therapeutic benefits, such as pain reduction and skin lesion improvement.

**Figure 4:**
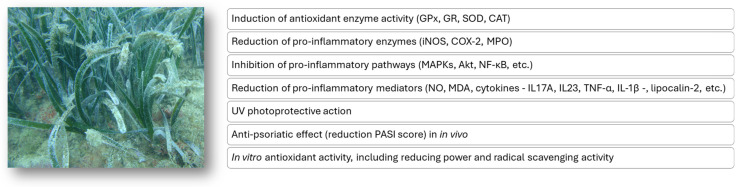
Putative mechanisms of the antioxidant and anti-inflammatory action of extracts from the *P. oceanica* marine plant.

The potential antioxidant and anti-inflammatory mechanisms of action attributed to *P. oceanica* extracts described in this review are overall schematically reported in Figure 4.

In conclusion, the scientific research summarized in Table 1 underscores the significant antioxidant and anti-inflammatory properties of *P. oceanica*. Traditionally valued for its therapeutic potential, actual scientific studies have confirmed its capacity to neutralize free radicals, reduce oxidative stress, and modulate inflammatory pathways. The bioactive compounds, particularly polyphenols, play a central role in these effects, with extract preparations showing promise in protecting skin cells, mitigating inflammatory responses, and potentially addressing chronic inflammatory conditions. Importantly, *P. oceanica* exhibits no notable toxicity both in vitro and in vivo, highlighting its safety profile for future therapeutic and cosmetic applications. Collectively, these findings position *P. oceanica* as a promising natural marine resource for developing novel strategies to prevent and treat oxidative stress-related and inflammatory diseases, warranting further exploration and clinical validation.

### 3.2. Cymodocea nodosa Bioactivity

The marine plant *C. nodosa* has emerged as a subject of interest in the realm of natural health due to its promising antioxidant and anti-inflammatory benefits. 

The antioxidant capacity of *C. nodosa* was first prominently documented by Kolsi et al. (2015). This study represented a significant innovation by thoroughly examining the chemical composition, biological properties, and potential nutritional benefits of a polysaccharide extracted from the leaves of the seagrass *C. nodosa* [71]. The authors isolated the sulphated polysaccharide from *C. nodosa* (CNSP) via hot water extraction and analyzed its chemical composition. The polysaccharide fraction was rich in sulphates and carbohydrates, while proteins and lipids were present in moderate and low amounts, respectively. Studies on CNSP in high-fat diet (HFD)-fed rats revealed significant antioxidant activity, evidenced by increased activities of SOD, CAT, and GPx, as well as enhanced reducing power—all of which were strongly dependent on concentration—and protecting the liver and kidneys of rats [71]. In 2017 Kolsi et al. revealed that CNSP exhibits a range of interesting properties, including antioxidant, antimicrobial, and cytotoxic activities, making it a promising candidate for future applications in nutraceuticals and functional foods, as well as in alternative medicine and natural therapies [72]. The results revealed that CNSP had high activity in the total antioxidant assay (59.03 mg AAE/g extract), reducing power (OD = 0.3), DPPH radical scavenging (IC_50_ = 1.22 mg/mL), and ABTS radical scavenging (IC_50_ = 1.14 mg/mL). Notably, CNSP demonstrated significant antioxidant activity in Hela cells subjected to oxidative stress induced by Fe_2_SO_4_ (100 mM). After oxidative stress induction, it was observed that Hela cells pre-treated with different concentrations of CNSP (0.015, 0.0035 and 0.0015 μg/mL for 72 h) showed a significant reduction in lipid oxidation. These findings open new opportunities for the use of CNSP in preventive and therapeutic strategies, laying the groundwork for future developments in the field of health and wellness [72].

The research underscores CNSP’s strong antioxidant capacity both in vitro and in vivo, highlighting its potential as a natural antioxidant agent for health promotion and disease prevention. These results align with the existing literature, as marine polysaccharides extracted from algae and marine organisms are attracting increasing interest for their potential anti-inflammatory and antioxidant properties [73,74,75].

In a further study by Kolsi et al. (2017), flavonoids and phenolic acid derivatives were characterized from a hydro-ethanolic extract of *C. nodosa* (CNE). Specifically, four flavonoids (catechin, quercetin-3-O-rutinoside, quercetin-3-O-glucoside, and isorhamnetin-3-O-rutinoside) and three phenolic acid derivatives (sinapinic acid, ferulic acid, and cinnamic acid) were characterized from the CNE. The CNE (100–2000 mg/kg body weight) demonstrated the ability to enhance antioxidant defenses by increasing levels of SOD, CAT, and GPx in the pancreas, liver, and kidneys of alloxan-induced diabetic rats. Additionally, it reduced lipid peroxidation (LPO) in these organs. Histological analyses confirmed tissue protection and regeneration [76]. This highlights how these bioactive compounds can bolster antioxidant defenses by increasing antioxidant enzymes in vital organs, thereby reducing oxidative stress and lipid peroxidation. The findings also suggest tissue protection and regenerative effects, which are significant for developing natural interventions for diabetes-related oxidative damage and organ protection.

In a related study, Chaabani et al. (2024) investigated the efficacy of ultrasound-assisted extraction techniques to maximize the recovery of phenolic compounds from *C. nodosa* [77]. The results revealed that, under optimized extraction conditions, both the total phenolic content (113.07 mg GAE/g dry extract) and antioxidant activity (67.02%)—assessed by a DPPH assay—of the extracts obtained were significantly high. In particular, the study identified synaptic acid, myricetin, and quercetin-3-O-rutinoside, well documented for their potent antioxidant and anti-inflammatory properties, as key phenolic compounds. In addition, the authors demonstrated the anti-inflammatory activity of *C. nodosa* extracts by measuring their ability to inhibit the production of NO, a recognized biomarker of inflammation, in RAW264.7 murine macrophage cells stimulated with LPS. A slight inhibition of NO production was observed at a non-cytotoxic dose of 400 µg/mL of *C. nodosa* extracts. The combined antioxidant and anti-inflammatory activity of the tested eco-friendly extracts suggests that they could be used in skincare cream to combat oxidative stress and skin inflammation [77]. This study provides scientific validation for the potential use of *C. nodosa*-derived phenolic-rich extracts as natural, eco-friendly ingredients in skincare products targeting oxidative damage and inflammation, aligning with current trends favoring plant-based and sustainable formulations.

**Figure 5:**
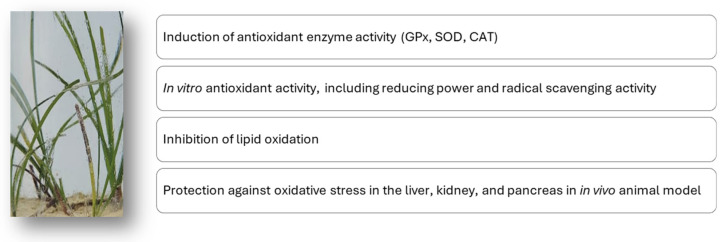
Putative mechanisms of the antioxidant and anti-inflammatory action of extracts from the *C. nodosa* marine plant.

The potential antioxidant and anti-inflammatory mechanisms of action attributed to *C. nodosa* extracts described in this review are overall schematically reported in Figure 5.

In conclusion, *C. nodosa* has demonstrated significant potential as a natural marine source with antioxidant and anti-inflammatory properties (Table 2). The reviewed studies reveal that its bioactive polysaccharides and phenolic constituents possess robust free radical scavenging, reducing, and anti-inflammatory activities, which could be harnessed in the development of functional foods, nutraceuticals, and topical formulations. Future research should focus on further elucidating its mechanisms of action, optimizing extraction techniques, and evaluating its efficacy and safety in clinical settings.

### 3.3. Zostera marina and Zostera noltii Bioactivity

The marine plant *Z. marina* has garnered significant attention in recent years due to its remarkable therapeutic properties, particularly in the domains of skin health and anti-aging.

A study indicates that hydroalcoholic extracts of *Z. marina* leaves possess substantial antioxidant activity and the ability to inhibit matrix metalloproteinase-1 (MMP-1) [78]. MMPs are the primary enzymes responsible for extracellular matrix breakdown; particularly, MMP-1 is a crucial enzyme in collagen degradation and skin aging processes. Inhibiting MMPs is crucial for maintaining youthful skin [79]. The authors investigated the role of three major compounds isolated from an ethyl acetate-soluble fraction of *Z. marina*, namely apigenin-7-O-β-d-glucoside, chrysoeriol, and luteolin. These compounds demonstrated significant antioxidant activity. Their effectiveness was quantified through SC_50_ values in DPPH assay and in xanthine/xanthine oxidase system, with luteolin showing the strongest activity (0.01 mM). Additionally, luteolin showed the most potent radical scavenging activity, demonstrating a remarkable ability to inhibit MMP-1 expression by up to 44% at 4 μM in a UVA-irradiated human skin fibroblast (Hs68) cell model. This effect is particularly significant, as it is directly related to the preservation of collagen integrity in the skin. In addition, luteolin was also found to suppress the production of IL-6 by 30% at 4 μM, which is a cytokine that promotes MMP-1 expression, in a UVB-irradiated human (HaCaT) keratinocyte cell model. These results further consolidate the role of *Z. marina* extracts as a potential photoprotective agent given its strong antioxidant activity [78]. This study underscores the relevance of *Z. marina* extracts as potent natural agents capable of combating skin aging processes through antioxidant activity and enzyme inhibition, highlighting their potential in skincare formulations aimed at photoprotection and anti-aging.

Free radicals are often the cause of LPO, and they are therefore responsible for the destruction of biological membranes; therefore, the use of antioxidant agents could ensure the protection of tissues against the effect of free radicals. In the work of Khasina et al. (2003), the authors induced LPO in a murine model using white male mice and evaluated the MDA liver levels, as well as the activity of GR and GPx in the liver [80]. LPO activation was induced by lead acetate (20 mg/kg of body weight), tetrachloromethane (300 mg/kg), sodium nitrite (50 mg/kg), or a mixture of polychlorinated biphenyls (SOVOL; 5 mg/kg). All tested substances caused an increase in hepatic MDA levels by an average of 1.8–2.5 times and a decrease in the activity of GR and GPx by 31–54%. The study demonstrated that two weeks after intragastric administration of a low-esterified pectin, named pectin-zosterin, in the form of a 1% gel at a dose of 100 mg/kg—isolated from raw material of *Z. marina*—the pro-oxidant process induced by LPO in mice was suppressed. These results provided the first evidence that pectin-zosterin from *Z. marina* enhances antioxidant mechanisms. However, the mechanism of the in vivo antioxidant effect of pectin-zosterin has not yet been elucidated. The authors hypothesize an antioxidant effect associated with the absorption properties of low-esterified pectins [80].

A comparative study conducted by Kolenchenko et al. (2005) further reinforced the antioxidant activity of the low-esterified pectin extracted from *Z. marina* raw material. This research demonstrated that the pectin exhibited superior reducing activity compared to established antioxidant agents used in medicine, namely Mildronate and Emoxipin. This was assessed through the inhibition of Fe^2+^ ascorbate-induced oxidation of Tween 80 (sorbitan monooleate) to malonic dialdehyde [81]. The study revealed that increasing the pectin concentration tenfold from 0.1% to 1% (0.1% solution: 1.7 ± 0.3%; 0.5% solution: 5.3 ± 0.4%; 1% solution: 10.9 ± 0.6%) significantly enhanced its reducing activity, resulting in an average 4.1-fold increase in reduced iron content after 60 min. In contrast, Emoxipin and Mildronat showed only about a 1.9-fold increase under similar conditions. When assessing inhibitory effects, solutions of 1%, 0.5%, and 0.1% concentrations of pectin and Emoxipin demonstrated that pectin’s inhibiting activity was higher—53.4%, 67.5%, and 80.2%, respectively—compared to Emoxipin’s 72.2%, 60.1%, and 76.7%, all relative to Mildronat. Overall, the low-etherified pectin exhibits stronger reducing properties in vitro than the antioxidative drugs Mildronat and Emoxipin. Its capacity to inhibit Fe^2+^-induced ascorbate oxidation of Tween 80 to malonic dialdehyde was less effective than Mildronat but comparable to Emoxipin, indicating its potential as a potent antioxidant with notable reducing activity.

According to the studies by Khasina et al. (2003) and Kolenchenko et al. (2005), it has been shown that polysaccharides, such as low-esterified pectin, are significant because they can directly scavenge free radicals, inhibit lipid peroxidation, and enhance endogenous antioxidant enzymes. The efficacy demonstrated in these studies suggests potential therapeutic applications for preventing diseases related to oxidative damage, preserving membrane integrity, and maintaining cellular health.

In a study conducted by Choi et al. (2009), a methanol crude extract of *Z. marina,* organic solvent fractions (n-hexane, chloroform, ethyl acetate, n-butanol), and a water fraction were screened for antioxidant activity [82]. The crude extract of *Z. marine* contained total polyphenols of 204.63 μg GAE/mg dry extract and showed a dose-dependent DPPH inhibitory activity (from 0.1 to 20 mg/mL) ranging from 3.12 ± 0.75% to 90.55 ± 2.34% and a dose-response reducing power (from 0.1 to 20 mg/mL) ranging from 0.03 ± 0.00% to 1.28 ± 0.06%. The water fraction was the most abundant (around 60.45%), but it contained the lowest phenolic content (50.25 μg/mg) and exhibited minimal antioxidant activity (<10% DPPH scavenging), indicating limited phenolic presence. Conversely, the ethyl acetate fraction, richest in phenolics (968.50 μg/mg), showed the strongest antioxidant effects, achieving over 95% DPPH scavenging with an IC_50_ of 0.46 mg/mL, which is comparable to standard antioxidants (BHA and ascorbic acid). The n-butanol fraction also demonstrated high scavenging activity (91.27% DPPH). Reducing power assays revealed that the n-butanol fraction (0.1 mg/mL) had the highest electron-donating capacity at lower concentrations, with both the ethyl acetate and n-butanol fractions exhibiting enhanced reducing activity at higher concentrations,’ correlating with their phenolic richness. Overall, phenolic compounds are predominantly concentrated in semi-polar and non-polar fractions, such as ethyl acetate and n-butanol, which possess significant antioxidant activities, underscoring *Z. marina*’s potential as a natural source of bioactive antioxidants for functional applications [82]. This research enhances the understanding of the antioxidant potential of *Z. marina* extracts, emphasizing the importance of phenolic compounds concentrated in specific solvent fractions. It supports the exploration of *Z. marina* as a promising natural source for antioxidant agents that can be harnessed in various health and industrial applications to mitigate oxidative stress and associated disorders.

In addition to its antioxidant properties, the marine plant *Z. marina* has been studied for its anti-inflammatory effects. A research conducted in 2015 by Kim et al. showed that an ethanolic extract of *Z. marina*, called ZMEE, significantly reduced NO production and iNOS expression in LPS-induced RAW264.7 murine macrophages in a dose-dependent manner (0.1–100 µg/mL) [83]. Furthermore, ZMEE (0.1–100 µg/mL) was found to significantly inhibit the secretion of pro-inflammatory cytokines, including IL-6, IL-1β, and TNF-α. It also suppressed NF-κB activation and phosphorylation of mitogen-activated protein kinases (MAPKs) such as JNK, ERK, and p38 after treatment [83]. Furthermore, the authors demonstrated that topical administration of ZMEE (20 µL/ear) alleviated the oedema induced by 5% croton oil in the ears of IRC mice. Histological analyses of mouse ear tissues revealed a reduction in dermal thickness and a decrease in infiltrating mast cells. These results suggest an anti-inflammatory potential of *Z. marina* [83]. This information underscores the potential therapeutic applications of *Z. marina* as an anti-inflammatory agent, contributing to the broader understanding of marine plants’ pharmacological properties.

**Figure 6:**
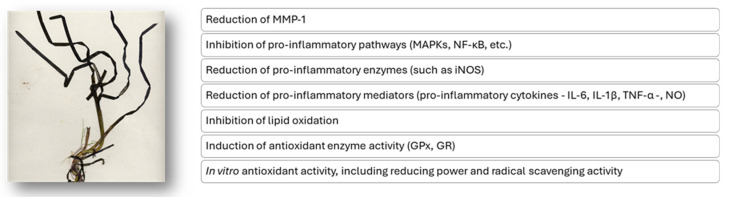
Putative mechanisms of the antioxidant and anti-inflammatory action of extracts from the *Z. marina* marine plant.

The potential antioxidant and anti-inflammatory mechanisms of action attributed to *Z. marina* extracts described in this review are overall schematically reported in Figure 6.

Belonging to the Zosteraceae family, *Z. noltii* shares a similar habitat with *Z. marina*, but presents significant differences in terms of bioactive properties and potential therapeutic applications. In a research conducted by Custódio et al. in 2014, a raw material methanol extract of *Z. marina*, collected in southern Portugal, showed a significantly higher total phenolic content (0.14 ± 0.01 mg GAE/g, dry weight) than that of *Z. noltii* (0.09 ± 0.00 mg GAE/g, dry weight). Rosmarinic acid, quantified by HPLC analysis, was found in the extracts for the two species and was the main component in *Z. marina* (0.24 mg/g dry weight), but not in *Z. noltei* (0.09 mg/g dry weight) [84]. This characteristic translates into a greater scavenging capacity for DPPH radicals (IC_50_ values of 0.31 ± 0.01 mg/mL and 1.10 ± 0.15 mg/mL in *Z. marina* and *Z. noltii,* respectively) and an effective copper chelation ability, suggesting potential therapeutic applications against oxidative stress. On the other hand, *Z. noltii* extract has shown lower antioxidant activity, although some capacity for chelation of metal ions has been identified. This difference in antioxidant activity may limit the therapeutic applications of *Z. noltii* compared to *Z. marina*. Overall, while *Z. marina* stands out for its promising antioxidant properties, *Z. noltii* shows significant limitations in these areas.

In conclusion, *Z. marina* exhibits a promising antioxidant and anti-inflammatory activities, which are primarily attributed to its rich content of phenolic compounds, summarized in Table 3. These constituents demonstrate significant radical scavenging capabilities, inhibition of matrix metalloproteinases involved in skin aging, and suppression of pro-inflammatory mediators, supporting its potential as a natural agent for skin health and photoprotection. Furthermore, the *Z. marina* ability to enhance endogenous antioxidant defenses underscores its capacity to mitigate oxidative stress at the tissue level. Comparative studies also highlight the superior antioxidant properties of *Z. marina* over related species like *Z. noltii*, emphasizing its therapeutic potential.

## 4. Bioactive Compounds from the Native Mediterranean Seagrasses and Their Therapeutic Potential

The ecological functions of seagrasses, such as providing protection against environmental stressors through the production of antioxidant and anti-inflammatory compounds, are directly related to their potential as sources of natural bioactive substances for human health [85]. The same secondary metabolites that help seagrasses defend against abiotic and oxidative stress also exhibit potent antioxidant and anti-inflammatory activities beneficial to human health, as previously discussed.

The work collected in this review clearly shows that native Mediterranean seagrasses are a rich source of secondary metabolites. In particular, it has been shown that the seagrass extracts described here are mainly composed of polyphenols, which have been attributed antioxidant and anti-inflammatory functions.

The main polyphenols identified in these seagrass extracts include chicoric acid, gallic acid, chlorogenic acid, catechin, epicatechin, ferulic acid, synaptic acid, myricetin, quercetin-3-O-rutinoside, quercetin, luteolin, apigenin-7-O-β-d-glucoside, chrysoeriol, quercetin-3-O-rutinoside, quercetin-3-O-glucoside, quercetin-3-O-malonylglucoside, isorhamnetin-3-O-rutinoside, isorhamnetin-3-O-glucoside, isorhamnetin-3-O-malonylglucoside, sinapinic acid, cinnamic acid, rosmarinic acid, procyanidin C2, and procyanidin B2.

It is important to emphasize that the bioactive properties of the seagrasses described have mainly been attributed to polyphenol-rich extracts. In particular, these extracts act as a phytocomplex, and their antioxidant and anti-inflammatory properties cannot be attributed to a single specific compound.

This aspect underlines the importance of considering the phytocomplex rather than the single compound, since the phytocomplex represents the entirety and abundance of the many secondary metabolites of the specific seagrass.

Among secondary metabolites, polysaccharides have also been extracted, such as the sulfated polysaccharide from *C. nodosa* (CNSP) and low-esterified pectin from *Z. marina*. These polysaccharides have demonstrated the ability to neutralize free radicals and reduce oxidative stress. According to the literature, it was observed that the chemical antioxidant activity of polysaccharides in vitro is greatly influenced by factors such as their solubility, sugar ring configuration, molecular weight, presence of charged groups, protein components, and covalently attached phenolic compounds [86].

It should be noted that the studies collected in this review describe the antioxidant and anti-inflammatory activities of seagrass extracts obtained through a wide variety of extraction methods. Variations in extraction protocols can influence the yield, potency, and composition of the bioactive compounds, thereby limiting the ability to draw definitive conclusions. Furthermore, phytochemical analysis was not conducted for all tested and reported extracts, making detailed comparative analysis difficult. This gap hampers the ability to identify specific active compounds responsible for the observed biological activities and restricts detailed mechanistic understanding. Additionally, when phytochemical characterization was performed, it was observed that the chemical composition varies depending on several factors, such as collection site, seasonality, depth, harvesting period, and others. In addition, most of the studies conducted so far are in vitro or preliminary in vivo, and there is therefore a paucity of clinical investigations to substantiate the health claims of compounds derived from seagrasses.

In summary, while the collected studies support the promising role of seagrass secondary metabolites in promoting human health, these limitations highlight the need for more standardized, comprehensive, and mechanistically oriented research to fully elucidate their potential and facilitate clinical translation.

## 5. Research Methodology

This overview collected 18 research articles found in the literature from 2003 to 2024 on the description of antioxidant and anti-inflammatory properties of native Mediterranean plants. Extensive searches were performed in PubMed, ScienceDirect, Web of Science, and Google Scholar databases using one or a combination of the following terms: “marine plants”, “seagrasses”, “marine natural compounds”, “marine plants with antioxidant properties”, “marine plants with anti-inflammatory properties”, “*Posidonia oceanica* bioactivity”, “*Cymodocea nodosa* bioactivity”, “*Zostera marina* bioactivity”, and “*Zostera noltii* bioactivity”. The marine plant images were provided by the staff of the Interuniversity Center of Marine Biology and Applied Ecology “G. Bacci” (CIBM) of Leghorn (Italy) and by Prof. Valentino Casolo of the University of Udine (Italy). Figures were created by Microsoft PowerPoint per Microsoft 365 MSO (Version 2502 Build 18526.20168).

## 6. Future Perspectives

With the increasing awareness of the importance of human health and well-being, seagrasses are emerging as a promising resource capable of providing innovative and sustainable solutions to address public health and environmental conservation challenges [85]. Integrating these resources into natural treatments could represent a significant step towards a holistic approach to health that recognizes the deep connection between humans and the marine environment.

Native Mediterranean seagrasses, in particular, offer a unique opportunity to improve human health, promote environmental sustainability, and stimulate innovation. These species, often adapted to specific conditions of the marine ecosystem, can provide bioactive compounds with potential health benefits, such as antioxidant and anti-inflammatory properties. The growing interest in the sustainable use of marine resources can also contribute to the protection of these delicate ecosystems, ensuring the availability of such resources for future generations.

NSAIDs are among the most well-known and widely used medications in both human and veterinary medicine. However, their widespread use has led to their identification as emerging contaminants in the environment. For example, in recent decades, the use of NSAIDs in companion animals has significantly increased, becoming a standard practice. In this context, the veterinary community must look to human studies as a crucial source of information to gain insights into the potential of new natural drugs that are free from side effects. Given the increasing abuse of NSAIDs in both human and veterinary medicine, this collection of scientific data, aligned with the “One Health” approach, highlights the anti-inflammatory potential of seagrasses and aims to serve and guide future research in this area [16].

We hope that the research compiled in this overview can stimulate a greater understanding of the potential of native Mediterranean seagrass and encourage the development of new strategies for the management and conservation of marine biodiversity, promoting a more sustainable and healthier future for all.

## Figures and Tables

**Table 1:** Experimental details of scientific research on the antioxidant and anti-inflammatory properties of *P. oceanica.*

Plant Material	Extraction Method	Compound Class	Cellular Model	Animal Model	In Vitro Assay	Ref.
Leaves	Hydro-ethanolic	Polyphenols		Wistar albino rats (150–250 mg/kg b.w.)		[63]
Leaves	Hydro-ethanolic (Gd-E4)	Polyphenols	Human skin HS-68 fibroblasts (0.15–1.5 µg/mL)		DPPH (IC_50_ 0.090 µg/mL)	[64]
Leaves	Hydro-ethanolic (PEE)	Polyphenols			DPPH (IC_50_ 32 ± 2 μg/mL)	[65]
Leaves	Hydro-ethanolic (PO)	Polyphenols			DPPH (IC_50_ 72.42 ± 22.9 mg/L)	[66]
Leaves	Methanolic (LP)	Polyphenols			DPPH (IC_50_ 8.2 ± 0.8 μg/mL); ABTS (IC_50_ 1.6 ± 0.9 μg/mL); hydroxyl radical scavenging (IC_50_ 267 μg/mL); superoxide anion radical scavenging (IC_50_ 71.0 ± 2.0 μg/mL); reducing power (19.5 ± 0.6 mg/mL)	[67]
Leaves	Hydro-ethanolic (POE)	Polyphenols	Murine RAW 264.7 macrophages (2.88 µg GAE/mL)		DPPH (11.0 ± 0.7 mg AAE/mL); FRAP (0.9 ± 0.2 mg AAE/mL)	[68]
Leaves	Hydro-ethanolic (POE)	Polyphenols		CD-1 mice (10–100 mg/kg b.w.)	DPPH (1.2 ± 0.04 mg AAE/mL); FRAP (0.24 ± 0.05 mg AAE/mL)	[69]
Leaves	Hydro-ethanolic (POE)	Polyphenols		C57BL/6 mice (100 mg/kg b.w.)	DPPH (1.1 ± 0.2 mg AAE/mL); FRAP (0.13 ± 0.07 mg AAE/mL)	[70]

**Table 2:** Experimental details of scientific research on the antioxidant and anti-inflammatory properties of *C. nodosa.*

Plant Material	Extraction Method	Compound Class	Cellular Model	Animal Model	In vitro Assay	Ref.
Raw material (CNSP)	Hot water	Polysaccharides		HDF-rats		[71]
Leaves (CNSP)	Water with ethanol precipitation	Polysaccharides	Epithelial cervical Hela cells (0.015–0.0015 μg/mL)		DPPH (IC_50_ = 1.22 mg/mL); ABTS (IC_50_ = 1.14 mg/mL); total antioxidant (59.0 mg AAE/g); reducing power (OD = 0.3)	[72]
Raw material (CNE)	Hydro-ethanolic	Pholyphenols		Male Wistar rats (100–2000 mg/kg b.w.)		[76]
Raw material	Hydro-ethanolic, ultrasound assisted	Polyphenols	Murine RAW 264.7 macrophage (400 µg/mL)		Total antioxidant (113.07 mg GAE/g); DPPH (67.02%)	[77]

**Table 3:** Experimental details of scientific research on the antioxidant and anti-inflammatory properties of *Z. marina* and *Z. noltii.*

Plant Material	Extraction Method	Compound Class	Cellular Model	Animal Model	In Vitro Assay	Ref.
Leaves (*Z. marina*)	Hydro-ethanolic	Polyphenols (luteolin)	Human skin Hs68 fibroblasts and human HaCaT keratinocytes (4 μM)		DPPH (0.01 mM); xanthine/xanthine oxidase system (0.01 mM).	[78]
Raw material (*Z. marina*)	Ammonium oxalate with ethanol precipitation	Polysaccharide (pectin-zosterin)		White male mice (100 mg/kg b.w.)		[80]
Raw material (*Z. marina*)	Ammonium oxalate with ethanol precipitation	Polysaccharide (pectin)			Fe^2+^ ascorbate-induced oxidation (0.1, 0.5, 1%; solution: 1.7 ± 0.3%; 5.3 ± 0.4%; 10.9 ± 0.6%)	[81]
Raw material (*Z. marina*)	Methanol (crude extract)	Polyphenols			DPPH (0.1–20 mg/mL: 3.12 ± 0.75%–90.55 ± 2.34%); IC_50_ = 0.46 mg/mL); FRAP (0.1–20 mg/mL: 0.03 ± 0.00%–1.28 ± 0.06)	[82]
Raw material (*Z. marina*)	Hydro-ethanolic (ZMEE)	Undescribed	Murine RAW264.7 cells macrophages (0.1–100 µg/mL)	IRC mice (20 µL/ear)		[83]
Raw material (*Z. marina*)	Methanol	Polyphenols			DPPH (IC_50_ = 0.31 ± 0.01 mg/mL)	[84]
Raw material *(Z. noltii)*	Methanol	Polyphenols			DPPH (IC_50_ = 1.10 ± 0.15 mg/mL)	[84]
